# Clinical measurement of the dart throwing motion of the wrist: variability, accuracy and correction

**DOI:** 10.1177/1753193418773329

**Published:** 2018-05-12

**Authors:** Vasiliki Vardakastani, Hannah Bell, Sarah Mee, Gavin Brigstocke, Angela E. Kedgley

**Affiliations:** 1 Department of Bioengineering, Imperial College London, London, UK; 2 Hand Therapy Department, Chelsea and Westminster Hospital, London, UK; 3 Department of Orthopaedic Surgery, Frimley Park Hospital, Surrey, UK

**Keywords:** Dart throwing motion, wrist, goniometry, kinematics, clinical assessment

## Abstract

Despite being functionally important, the dart throwing motion is difficult to assess accurately through goniometry. The objectives of this study were to describe a method for reliably quantifying the dart throwing motion using goniometric measurements within a healthy population. Wrist kinematics of 24 healthy participants were assessed using goniometry and optical motion tracking. Three wrist angles were measured at the starting and ending points of the motion: flexion–extension, radial–ulnar deviation and dart throwing motion angle. The orientation of the dart throwing motion plane relative to the flexion–extension axis ranged between 28° and 57° among the tested population. Plane orientations derived from optical motion capture differed from those calculated through goniometry by 25°. An equation to correct the estimation of the plane from goniometry measurements was derived. This was applied and differences in the orientation of the plane were reduced to non-significant levels, enabling the dart throwing motion to be measured using goniometry alone.

## Introduction

Wrist motion has traditionally been analysed using two orthogonal axes of rotation: flexion–extension (FE) and radial–ulnar deviation (RUD) (Volz et al., 1980). However, recent studies report that while performing many activities of daily living, the wrist articulates about an oblique axis defined by the dart throwing motion (DTM) (Crisco et al., 2005; Garcia-Elias., 2008; Leventhal et al., 2010; Wolfe et al., 2006).

DTM is predominantly the result of midcarpal joint movement (Bugden, 2013; Crisco et al., 2005; Leventhal et al., 2010; Li et al., 2005; Moojen et al., 2003). This suggests that motion in the DTM plane can be preserved following wrist surgery, such as radiocarpal fusion, provided that mid-carpal motion is permitted (Arimitsu et al., 2009; Calfee et al., 2008; Moritomo et al., 2014). DTM has been shown to allow minimal relative rotation of the scaphoid and lunate (Crisco et al., 2005; Edirisinghe et al., 2014; Moritomo et al., 2014; Werner et al., 2004). Therefore, DTM may be permitted during early mobilization following injury. However, an accurate measurement of the DTM is required before it is used in clinical practice, since deviation from the DTM path has been shown to increase the relative motion of the carpal bones (Crisco et al., 2005).

Therefore, the aim of this study was to determine a method for reliably describing the DTM using goniometric measurements within a healthy population. Our approach consisted of using optical motion capture technology in conjunction with standard clinical tools to propose an accurate clinical measurement protocol for DTM.

## Methods

### Subjects

Following approval from our institutional research ethics committee (ICREC reference: 15IC2637), 24 healthy participants (15 men, 9 women, mean age 27 years (SD 4 years), weighing 69 kg (SD 13 kg) and 1.72 m tall (SD 0.10 m)) were recruited for this study. Informed consent was obtained from all participants prior to their enrolment. Participants were included if they had no history of osteoarthritis, activity limiting pain or injury, or surgical intervention in their wrists. Since this study aimed to investigate DTM in a healthy population, cases of generalized joint laxity (hypermobility) were excluded, as they present increased ranges of joint motion (Soucie et al., 2011). Potential participants were tested for hypermobility syndrome using the Beighton scale, with an applied cut-off level ≥4/9 (Beighton et al., 1973). To assess hand dominance, each participant was asked to complete the Edinburgh Handedness Survey (Oldfield, 1971). Both the dominant and non-dominant hands of the participants were tested.

### Optical motion capture protocol

**Figure 1.**
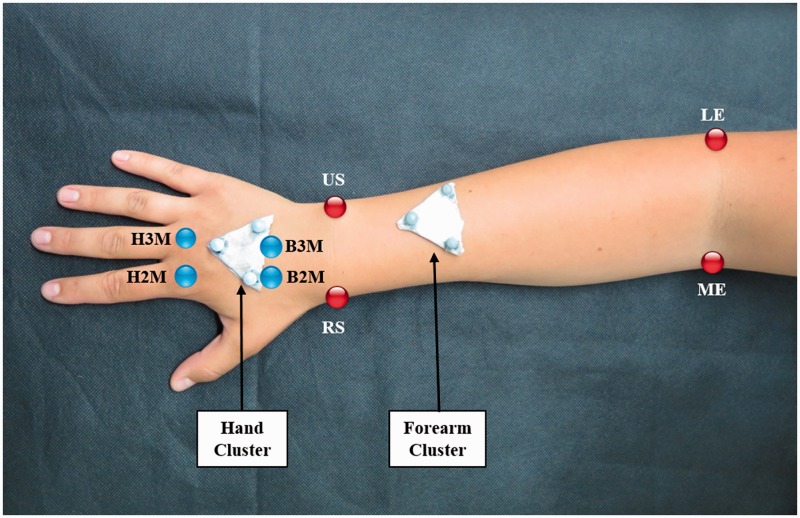
The configuration of the clusters of reflective markers placed on the hand and forearm, along with the digitized landmarks. The digitized landmarks of the hand, the head (H2M) and base (B2M) of the 2nd metacarpal and head (H3M) and base (B3M) of the 3rd metacarpal, are shown in blue, and those of the forearm, the radial (RS) and ulnar (US) styloids and lateral (LE) and medial (ME) epicondyles, are shown in red.

A 6-camera optical motion capture system (Qualisys AB, Gothenburg, Sweden) was used to measure wrist kinematics of the cohort (Appendix A, available online). To recreate DTM, participants were asked to hold a pen as they would hold a dart (Edirisinghe et al., 2014) and perform the motion, ending with release of the object. Each participant was asked to perform 15 repetitions of DTM. A 30-second rest period was designated between sets of five repetitions, in order to avoid the effects of fatigue. To track three-dimensional kinematics, clusters of reflective markers were placed on both hands and forearms of the participants (Figure 1). Using a calibrated stylus, the position of anatomical landmarks of the hand and forearm (Figure 1) were located relative to the respective cluster (Wu et al., 2005). The coordinate system of the hand was defined according to recommendations by the International Society of Biomechanics (Wu et al., 2005). For the forearm, the midpoints between the radial and the ulnar styloids and the lateral and medial epicondyles were used for the coordinate frames in order to facilitate comparisons between motion capture and goniometry measurements. Joint angles for the wrist were calculated using Euler angles (three angles describing the orientation of a rigid body with respect to a fixed coordinate system) with the International Society of Biomechanics rotation sequence using custom-written MATLAB (R2014b, Mathworks Inc., Natick, MA, USA) code.

### Clinical measurement protocol

**Figure 2.**
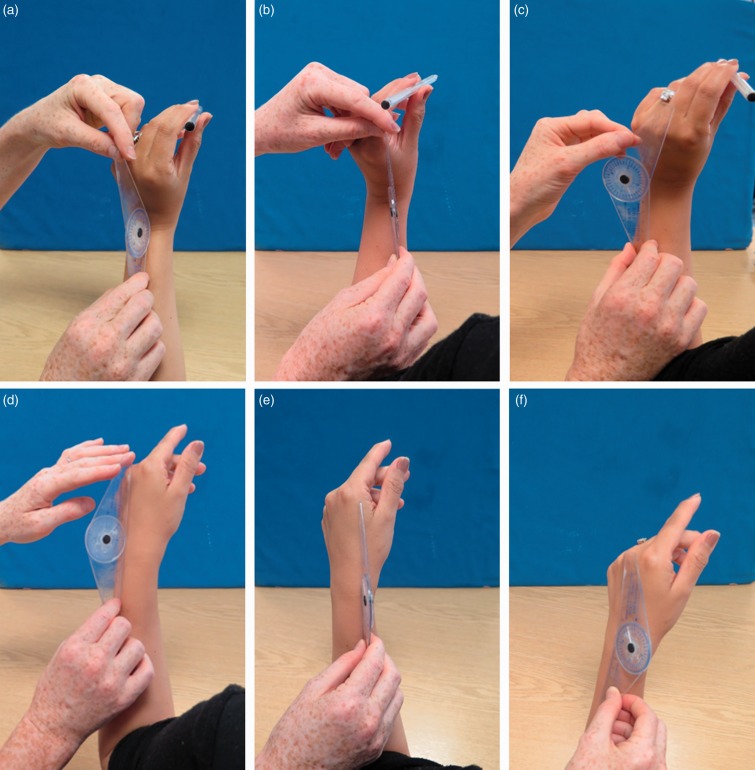
Goniometry measurements. At the start of the motion: (a) extension, (b) DTM and (c) radial deviation angle. At the end of the motion: (d) flexion, (e) DTM and (f) ulnar deviation angle. For measurements of the DTM angle, the arms of goniometer were aligned with the dorsal side of the radius and the second metacarpal. For all other measurements, the goniometer was placed according to standard clinical practice.

To quantify the DTM using goniometry, each participant was asked to hold a pen and perform the motion in the same way as instructed for the optical motion capture trials. One consultant hand therapist and one senior physiotherapist in hand therapy measured the angles of motion at the extremes of the range using a goniometer (Promedics, Blackburn, UK). Measurements were taken at the start and end of the composite DTM. These included pure FE and RUD angles as well as DTM measurements with combined extension and radial deviation followed by flexion and ulnar deviation (Figure 2). In order to obtain FE and RUD angle measurements, the arms of the goniometer were aligned with the third metacarpal and the midpoint of the forearm. However, for DTM range of motion measurements, the arms of the goniometer were aligned with the second metacarpal and the dorsal side of the radius (Bugden, 2013). Each measurement was performed twice in each session.

In order to assess repeatability and accuracy of the measurement of the angles, the mean of the goniometry measurements of each hand therapist for each participant was compared with the respective mean of the motion capture measurements. For this purpose, motion capture measurements were isolated at the beginning and the end of the DTM for each trial. To quantify the repeatability of the DTM, 20 participants returned for a second session after 1 week, repeating both the optical motion capture and clinical measurement protocols.

### Quantifying the DTM plane

To estimate the DTM plane parameters, a robust regression analysis was used. A Theil–Sen estimator (Sen, 1968) was applied to motion capture and goniometry results to estimate the slope and intercept of the DTM plane as a function of FE and RUD (see Appendix B, available online). The slope describes how much RUD occurs for a given amount of FE and the intercept describes how much RUD is present at neutral FE. For the motion capture results, the Theil–Sen estimator was fit simultaneously using all 15 repetitions of each session. For the goniometry results, one regression analysis was performed for each hand therapist; all measurements were included. All data analysis and robust regression were performed in MATLAB.

### Goniometry correction

Using the results of the motion capture system, a mathematical correction was designed in order to improve the accuracy of the clinical measurements. The equations used for this correction were based on both the anatomical characteristics of the wrist and the nature of the DTM. The aim of the method described below is to improve goniometry measurements of DTM in clinical practice, rather than introducing a new measurement instrument.

During DTM, the wrist performs a planar motion (Crisco et al., 2005; Moojen et al., 2003; Moritomo et al., 2014). As shown in previous studies (Brigstocke et al., 2014; Moojen et al., 2003), a linear relationship between FE and RUD angles may be applied for wrist motion along the DTM plane.

In clinical measurements of FE angles of the wrist, the third metacarpal is used as the guide for the moving arm of the goniometer and the radius as the guide for the stationary arm. Similarly, the DTM angle is measured using the second metacarpal and the radius as guides (Bugden, 2013). However, the second and the third metacarpal are commonly considered as a rigid segment (Bugden, 2013; Chao et al., 1989; El-Shennawy et al., 2001) and would be expected to exhibit similar motion during DTM.

**Figure 3.**
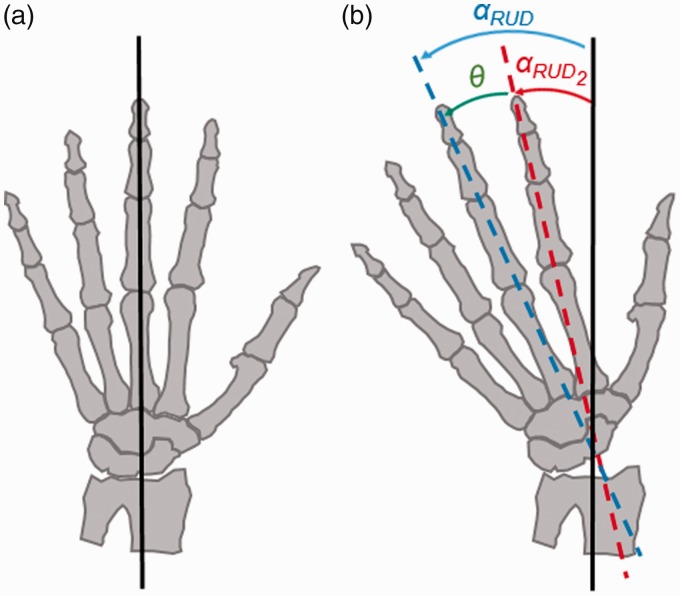
Radioulnar deviation angles of the second (RUD_2_) and the third (RUD) metacarpal. (a) The hand in a neutral position. (b) When the hand deviates from neutral (here, ulnar deviation), the deviation angle of the third metacarpal is assumed as the deviation angle of the second metacarpal plus the angle θ between the second and third metacarpals.

Additionally, due to the rigid connection between the two metacarpals, during the DTM the RUD angle of the wrist is correlated with the RUD component of the DTM angle in the coronal plane, as shown in Figure 3. Taking into account the anatomical considerations described above and the resulting mathematical relationships, described in Appendix B, a single equation was formed that describes the parameters of the DTM plane as a function of the FE and DTM angles:

$$\alpha_{\text{DTM}}=\frac{1}{{\text{C}}_{1}}\cdot [{\text{slope}}_{\text{DTM}}\cdot \alpha_{\text{FE}}+{\text{intercept}}_{\text{DTM}}-\theta -{\text{C}}_{2}]$$

where $\alpha_{\text{DTM}}$ and $\alpha_{\text{FE}}$ are the DTM and FE angles of the wrist measured by goniometry, θ is the angle between the second and the third metacarpals and C_1_ and C_2_ are constants calculated from the motion capture data.

The correction method can be divided into two steps. During the first step in fully defining the equation, the motion capture data are used to calculate parameters C_1_ and C_2_ so that the correction can be used with goniometric measurements. At this stage, no goniometry data are used. First, the motion capture data are used to calculate the angle between the vectors of the second and third metacarpals, θ. This was done using digitized points on the bones (head and base). Once the angle θ was defined, for each subject the slope and intercept of the Theil-Sen estimator were used as the slope (${\text{slope}}_{\text{DTM}}$) and intercept of the DTM plane $({\text{intercept}}_{\text{DTM}})$. The parameters C_1_ and C_2_ were defined for DTM using the optical motion capture measurements for the FE and DTM angles. The medians of C_1_ and C_2_ then were calculated for the tested population and the values were employed in the correction. Once these parameters are fully defined, the correction can be used directly on clinical data.

**Figure 4.**
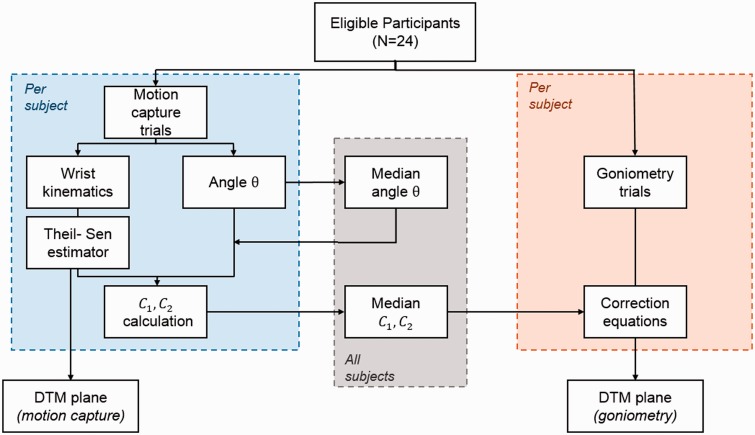
Flow chart of the development of the correction method. Motion capture data were used to calculate the angle θ and the correction constants (C_1_, C_2_) for each subject. Median values of the total cohort were calculated. These values, with angles measured with goniometry, were used to correct the DTM plane.

In the second step of the correction method, using the newly determined parameters C_1_ and C_2_ and the goniometry angles as inputs to the correction equation, the DTM plane orientation is defined. To make this correction subject specific, the angle θ was recalculated using the RUD angle at the end of the DTM. Therefore, the angle θ in this step was calculated from the measurements of the hand therapist. With a subject-specific θ, the correction was then used to calculate the DTM plane orientation based on the FE and DTM angles obtained by the hand therapists (Figure 4).

## Statistical analysis

All differences in orientation of the DTM plane were assessed using repeated measures analysis of variance (ANOVA), stratified by hand. Before applying any parametric statistical test, a Shapiro–Wilk test of normality was performed. Accuracy of the measurement of individual angles was estimated using intra-class correlation coefficients (ICCs, type (2,1) with absolute agreement) for the results of the two methods. Leave-one-out cross validation was performed in MATLAB to determine the accuracy of the estimate for the DTM plane parameters based on the motion capture measurements. Additional details regarding the statistical methods may be found in Appendix C, available online.

## Results

### Comparison between motion capture and goniometry

Significant differences were found when comparing the slopes derived from the motion capture data with those calculated based on goniometry (*p* < 0.001 for both dominant and non-dominant hands, separately). Goniometry measurements had a shallower slope with the mean difference between the two measurement techniques being 25° (SD 16°). Differences of 10° (SD 8°) were observed between the hand therapists (*p* < 0.001 for both dominant and non-dominant hands, separately).

The results of the comparison of each angle between the two methods using ICCs are reported in Table 1. The repeatability of most measurements indicated either good (0.61–0.80) or moderate (0.41–0.60) agreement (Altman, 1991). Lower ICC scores were found for radial deviation measurements. The comparisons between the measurements of the two hand therapists, quantified using inter-observer correlation coefficients, are presented in Table 2. All indicated good or very good (0.80–1.00) agreement, with the exception of extension of the non-dominant hand, which indicated fair (0.21–0.40) agreement (Altman, 1991).

**Table 1.** ICCs describing the comparison between hand therapist measurements (HT1 and HT2) and optical motion capture measurements.

Wrist motion	HT1	HT2
Flexion	0.55	0.60
Extension	0.65	0.63
Radial deviation	0.36	0.25
Ulnar deviation	0.60	0.45
DTM angle (start)	0.57	0.68
DTM angle (end)	0.61	0.59

**Table 2.** Inter-observer correlation coefficients describing the comparison between hand therapists for range of motion measurements.

Wrist motion	Non-dominant hand	Dominant hand
Flexion	0.72	0.63
Extension	0.21	0.51
Radial deviation	0.62	0.57
Ulnar deviation	0.68	0.74
DTM angle (start)	0.51	0.60
DTM angle (end)	0.73	0.82

### DTM plane definition and orientation

**Figure 5.**
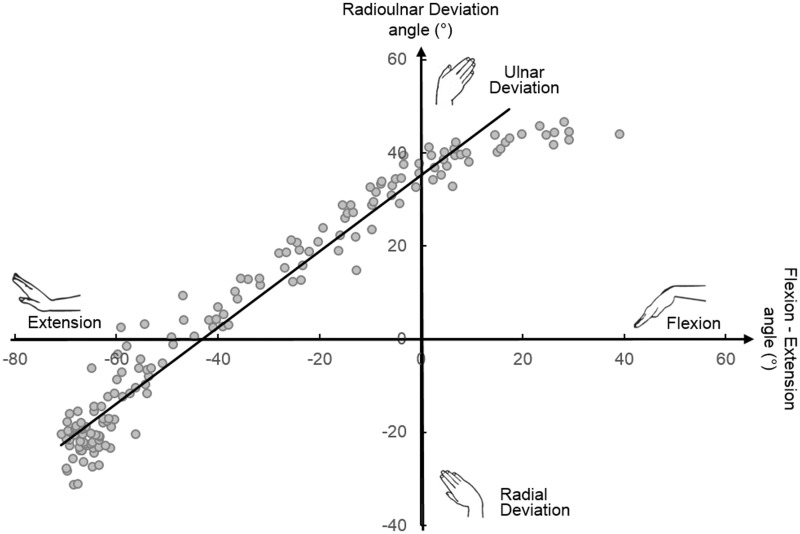
Theil-Sen estimator of the DTM plane (solid line) for the optical motion capture data of a single representative subject. The slope and intercept of the DTM plane can be visualized as the angle between the Theil-Sen estimation line and the FE axis and the intercept of the line and the radioulnar deviation axis, respectively.

Our regression analysis verified that the DTM is planar. The applied linear regression fitted the data well and the correlation coefficient was above 0.85 in all cases (Figure 5).

The slope of the DTM plane with respect to the FE axis in the transverse plane, as determined using Theil-Sen estimators (Figure 5), ranged between 28° and 57° among the tested population. The inter-subject variability could not be classified as a function of sex, age, ethnicity or hand dominance. Based on our measurements, the mean DTM plane was estimated to have an angle of 43° (SD 14°) to the FE axis. The intercept of the mean DTM plane indicated the wrist to be in 34° (SD 12°) ulnar deviation at neutral FE.

In order to assess the repeatability of the DTM, repeated measures ANOVA was conducted on all subjects that performed two optical motion capture sessions. No significant differences were observed in the slope of the plane between dominant and non-dominant hands. The mean absolute deviation of the plane orientation between the two sessions was 15° for both hands. However, the difference was not significant.

### Correction method: parameter calculation and application

In the first step, the median value of angle θ, based on motion capture data for all participants, was 7.3°. The medians of constants C_1_ and C_2_ were 0.79 and 16.61, respectively. Leave-one-out analysis showed low root-mean-square-errors for the estimates of both parameters $({\mathrm{RMSE}}_{c_{1}}=0.39^{\circ },{\mathrm{RMSE}}_{c_{2}}=0.21^{\circ })$.

In the second step, since ulnar deviation measurements were accurate when compared with motion capture data (Table 1), the pair of ulnar deviation and DTM angle measurements of the hand therapists, measured at the end of the motion, was used to calculate subject-specific angles between the second and third metacarpals.

**Figure 6.**
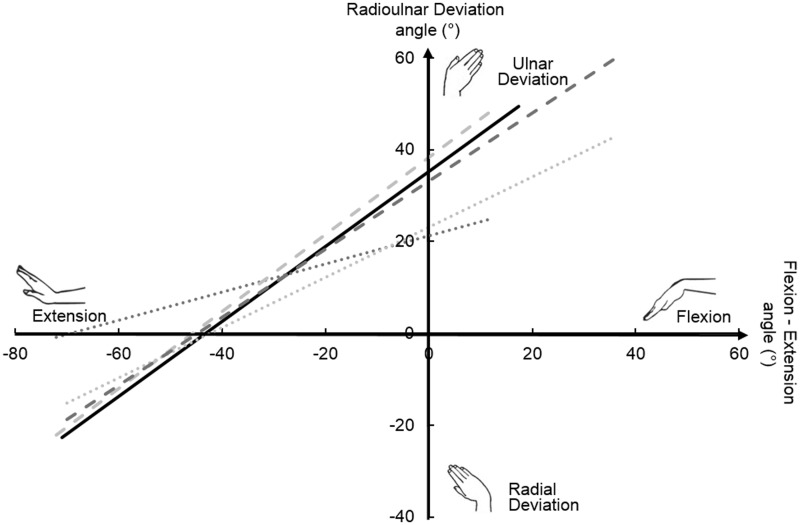
Dart throwing motion plane for a single representative subject measured by optical motion capture (solid line) and compared with the measurements of two hand therapists before (dotted lines) and after (dashed lines) the application of the correction method.

The derived method, using FE and DTM goniometry angles, reduced the mean absolute error between optical motion capture and goniometry estimations of the DTM plane. For both hand therapists, the mean absolute error of the slope of the DTM plane using the corrected goniometry data with respect to the one calculated from motion capture data was 12° (SD 7°). In both cases, the corrected DTM plane derived from goniometry was not significantly different from that derived from the optical motion capture (*p* ≥ 0.59 for HT1, *p* ≥ 0.19 for HT2 in both sessions) (Figure 6).

Therefore, the final correction can be summarized as the following:

$$\alpha_{\text{DTM}}=\frac{1}{0.79}\cdot [{\text{slope}}_{\text{DTM}}\cdot \alpha_{\text{FE}}+{\text{intercept}}_{\text{DTM}}-\theta -16.61]$$

The practical use of this mathematical correction in the clinical situation would be to use a combination of goniometric measurements at the start (pure extension and DTM angle measurements) and the end (pure flexion, ulnar deviation and DTM angle measurement) of DTM as the inputs to accurately estimate the orientation of the DTM plane.

## Discussion

In order to improve the estimation of the DTM plane using clinical tools, goniometry measurements of the angles were compared with those from optical motion capture. Differences between the two methods indicated that goniometry was not able to quantify DTM based on FE and RUD measurements. Lower ICC scores further supported the inability of the goniometry to produce accurate measurements. Radial deviation measurements exhibited highest errors. Goniometers have been reported to produce accurate measurements when the hand is positioned in either pure FE or pure RUD (Carter et al., 2009; Horger, 1990; LaStayo and Wheeler, 1994). However, the DTM is a composite motion, making it difficult to align the goniometer effectively (Bugden, 2013) in the radially extended position at the start of DTM. This explains the low ICC scores, not only describing the comparison between the two methods (Table 1), but also between the two hand therapists (Table 2). Without correction, the goniometry-based estimation of the DTM plane was dependent upon the hand therapist and differed from the gold standard estimation. However, when the correction was applied, the errors were reduced such that there were no significant differences with the plane estimated using optical motion capture.

Despite large inter-subject variability, the slope and intercept of the mean DTM plane, based on the motion capture data, were in accordance with those of previous studies (Brigstocke et al., 2014; Cliff and Rust, 2016; Moritomo et al., 2014). The relatively large range of DTM plane slopes indicates that using a mean DTM plane as a baseline for early rehabilitation is not likely to restrain wrist motion effectively along the subject-specific plane. Cadaveric and in vivo studies have demonstrated that both lunate and scaphoid motion is minimal during motion in the true DTM plane (Crisco et al., 2005; Moritomo et al., 2014; Werner et al., 2004). Early post-surgical rehabilitation of the wrist along a plane that is not consistent with the DTM may result in excessive scaphoid and lunate motion and compromise the surgical reconstruction. Accurate measurement of DTM in the clinic is therefore important for both clinical assessment and the use of DTM in the design of rehabilitation tools, such as dynamic splints and strengthening programmes that will allow mobilization of the wrist only along the DTM plane (Garcia-Elias, 2008). Early mobilization of the wrist is important to prevent the development of fibrosis and secondary stiffness, as well as regaining proprioception more quickly, ensuring a faster return to function.

The 15° mean absolute difference between the DTM plane estimation of the two sessions indicates that the wrist may be allowed to move in a range of functional DTM planes that are almost parallel, as shown previously (Moritomo et al., 2014). The observed variability can be attributed to the difficulty in ensuring a consistent plane of movement at the mid-carpal joint during active exercise in DTM, minimizing motion at the proximal carpal row. Although the dynamics of complex active motions like DTM change with proximal movement or position of the forearm, any constraint in the way the motion is performed would defeat the purpose of this method, which is to enable clinical assessment. Finally, the current practice of using the contralateral hand as a guide for the range of motion before injury or surgical intervention is further supported since no significant differences were found in the DTM plane between dominant and non-dominant hands.

In conclusion, although goniometry was initially not able to quantify the plane accurately, we developed a correction method that enables the measurement of this important functional motion to be used with confidence as part of clinical assessment and rehabilitation.

## Supplemental Material

Supplemental material for Clinical measurement of the dart throwing motion of the wrist: variability, accuracy and correction Click here for additional data file. Supplemental material for Clinical measurement of the dart throwing motion of the wrist: variability, accuracy and correction by Vasiliki Vardakastani, Hannah Bell, Sarah Mee, Gavin Brigstocke and Angela E. Kedgley in Journal of Hand Surgery (European Volume)

## Supplementary material

Appendices A, B and C are available at: journals.sagepub.com/doi/suppl/10.1177/1753193418773329.
