# Supplementary material for: Clinical measurement of the dart throwing motion of the wrist: variability, accuracy and correction
Source: J Hand Surg Eur Vol. 2018 May 12;43(7):723–31. doi: 10.1177/1753193418773329 (PMC6104201; doi:10.1177/1753193418773329)
Supplement: Supplemental material for Clinical measurement of the dart throwing motion of the wrist: variability, accuracy and correction [file Supplemental_material.pdf]

Clinical measurement of dart throwing motion of the wrist: variability, accuracy and correction

Vasiliki Vardakastani, Hannah Bell, Sarah Mee, Gavin Brigstocke, Angela E Kedgley

Appendix A: Motion capture measurement set-up

A 6-camera optical motion capture system (Qualisys AB, Gothenburg, Sweden) was used to measure wrist kinematics of the cohort (Fig. A1).

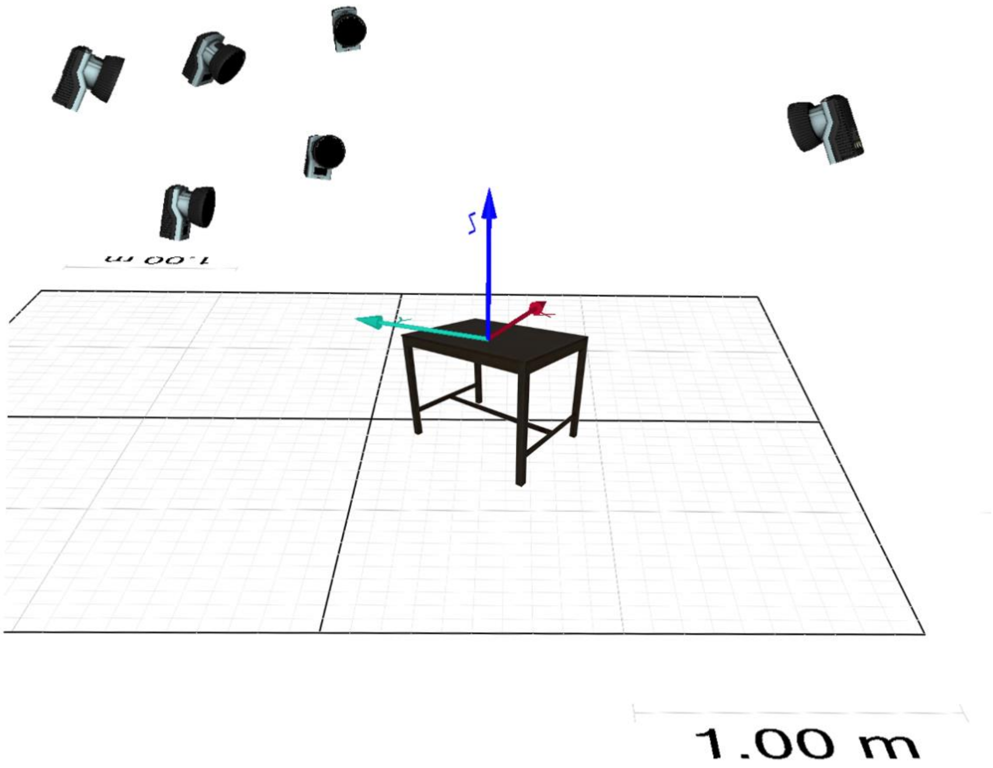

Figure A1. Motion capture setup visualised in QTM (Qualisys, Gothenburg, Sweden).

Appendix B: Mathematical description of the correction method

During the dart throwing motion (DTM) the wrist moves on an arc from radial extension to ulnar flexion, as shown in Figure B1.

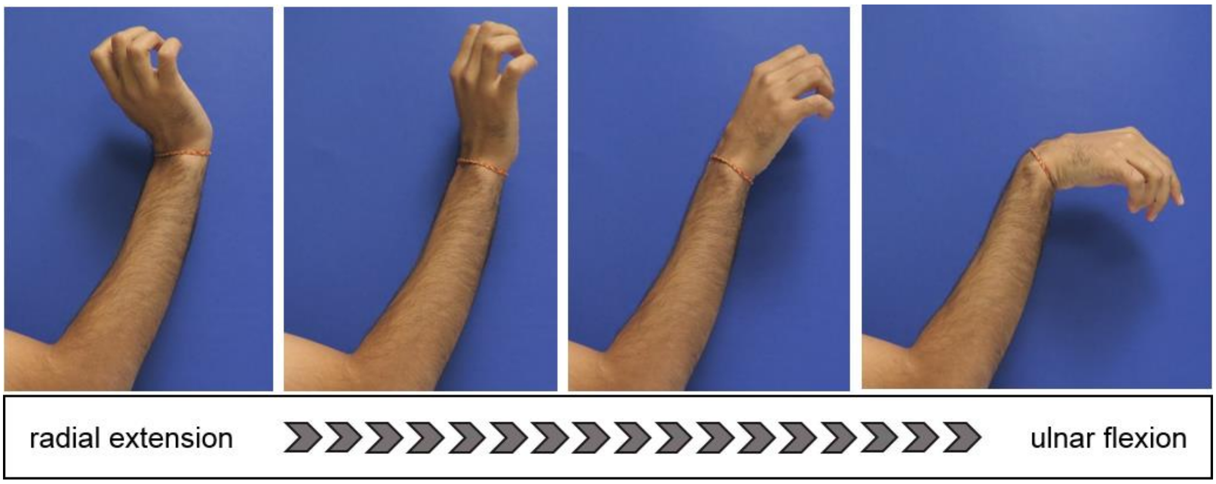

Figure B1. The dart throwing motion.

A linear relationship between flexion-extension (FE) and radial-ulnar deviation (RUD) angles, both measured as the angle between the radius and third metacarpal, may be applied for wrist motion in the DTM plane (Fig. B2):

$$\alpha_{RUD} = slope_{DTM} \cdot \alpha_{FE} + offset_{DTM} \qquad (1)$$

where α_{RUD} and α_{FE} are the RUD and FE angles of the wrist, respectively.

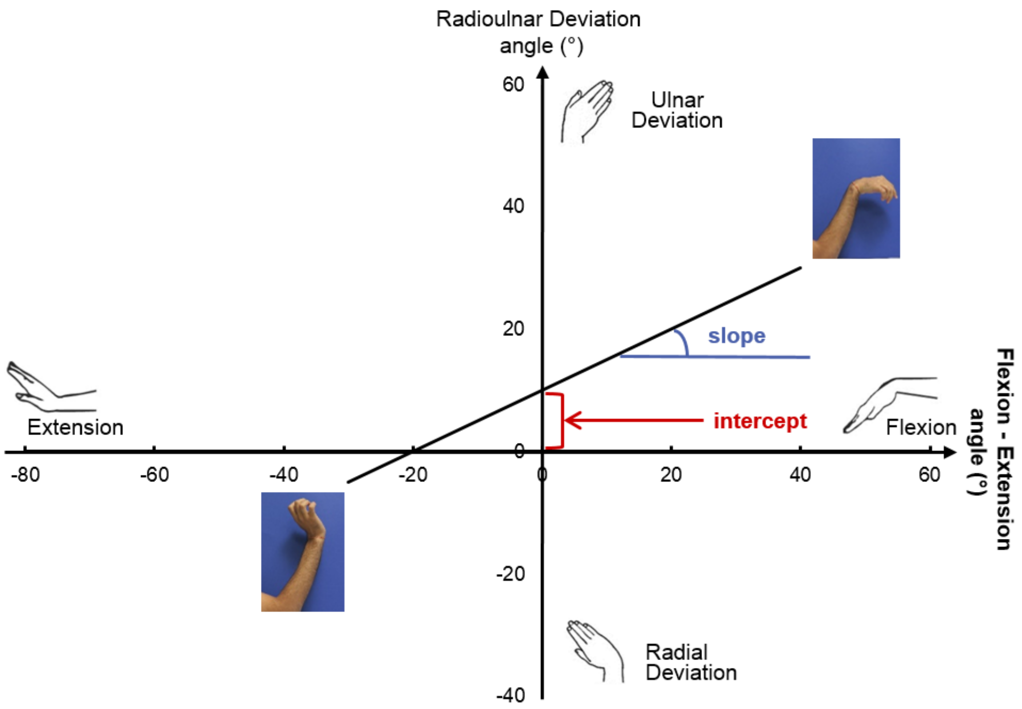

Figure B2. Visualisation of the slope and intercept parameters.

The second and third metacarpals are assumed to exhibit similar motions during DTM, due to the rigid connection between them. Therefore a second linear relationship was derived:

$$\alpha_{RUD_2} = C_1 \cdot \alpha_{DTM} + C_2 \qquad (2)$$

where α_{RUD_2} is the RUD angle of the second metacarpal, α_{DTM} is the measured DTM angle and C_1 and C_2 are the slope and intercept of the plane according to the second metacarpal reference frame (Fig. B3).

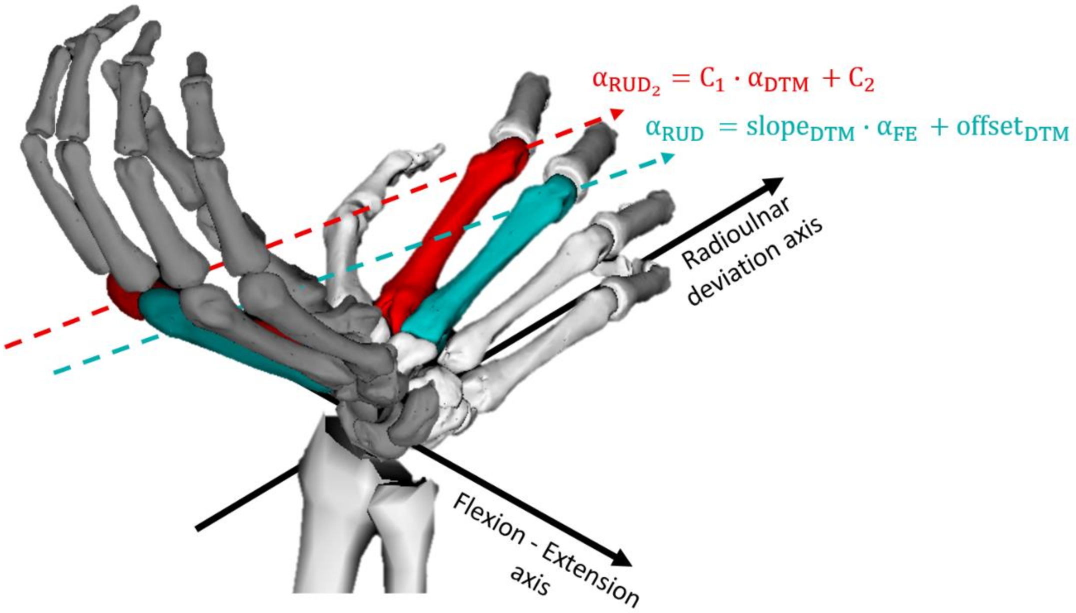

Figure B3. Visualisation of both equations on the wrist.

Based on the rigid connection between the two metacarpals, the RUD angle of the wrist can be correlated with the angle between the radius and the second metacarpal. Therefore, the RUD component of the DTM angle in the coronal plane (Figure 4) can be expressed as follows:

$$\alpha_{RUD_2} = \alpha_{RUD} - \theta \qquad (3)$$

where θ is the angle between the second and the third metacarpals.

Applying this restriction, the two relationships may be combined into one equation that describes the parameters of the DTM plane as a function of the FE and DTM angles:

$$\alpha_{DTM} = \frac{1}{C_1} \cdot [slope_{DTM} \cdot \alpha_{FE} + intercept_{DTM} - \theta - C_2] \qquad (4)$$

where C_1 and C_2 are calculated from the motion capture data.

Appendix C: Statistical Methods

In the following appendix, statistical methods presented in the paper are further explained.

Normality assessment

In order to assess the normality of the data before applying any parametric tests a Shapiro-Wilk test was used and non-significant results were acquired, indicating agreement with the normal distribution. Further to the statistical test, a graphical method was also used to further prove that our data follows the normal distribution (Figure C1).

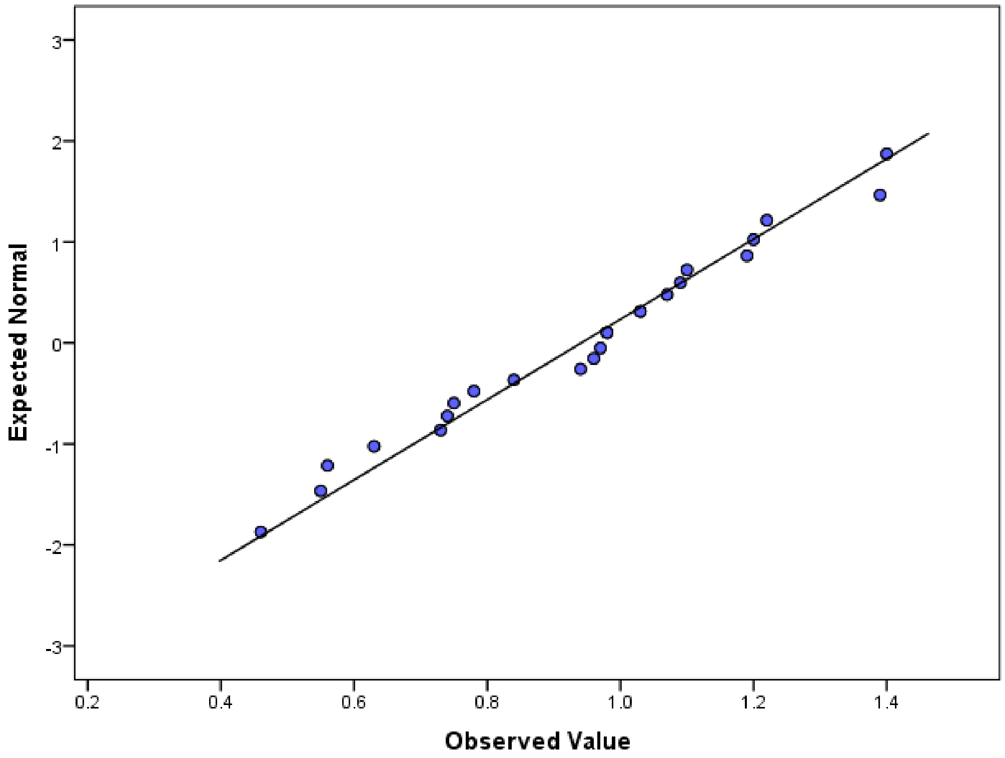

Figure C1. The normal Q-Q plot acquired shows normality of our data graphically.

Robust regression analysis

In this study, a Theil-Sen estimator was used in order to estimate the parameters of the DTM plane from the measurements acquired through the two methods. The Theil–Sen estimator is a method for fitting a line to a set of points (linear regression). In this method of linear regression, the regression model is selected based on the median of the slopes and intercepts of all pairs of data points. The main advantage of this method is that it is not as sensitive to outliers as other parametric methods and

maintains its accuracy in cases of non-normality or heteroscedasticity of the data. Three separate robust regressions were performed using the motion capture measurements and the measurements of each hand therapist, resulting in three different plane estimations.

Intra-class correlation coefficient (ICC) type (2,1)

An intra-class correlation coefficient (ICC) is a measure to determine how strongly values resemble one another. ICC type (2,1) with absolute agreement was used to assess the differences between the two measuring technique. A two-way random effect model was selected in order to detect similarities between the three groups of measurements: the motion capture measurements and the two groups of goniometry measurements (one for each hand therapist). An absolute agreement condition was applied to account for the possibility of systematic errors between different techniques.

Leave-one-out cross validation

Leave-one-out cross validation was used to validate the accuracy of model when estimating the plane parameters. In this case, both C_1 and C_2 parameters were calculated using motion capture data while omitting one measurement each time. The resulting deviations are reported in the manuscript.
